# Glutamate Attenuates the Survival Property of IGFR through NR2B Containing N-Methyl-D-aspartate Receptors in Cortical Neurons

**DOI:** 10.1155/2020/5173184

**Published:** 2020-08-11

**Authors:** Xia Zhao, Chao Han, Zhiwen Zeng, Linlin Liu, Haitao Wang, Jiangping Xu, Zhong-Ping Feng, Peter J. Little, Remi Quirion, Wenhua Zheng

**Affiliations:** ^1^Faculty of Health Sciences, University of Macau, Taipa, Macau, China; ^2^State Key Laboratory of Ophthalmology, Zhongshan Ophthalmic Center and School of Pharmaceutical Sciences, Sun Yat-sen University, Guangzhou, China; ^3^School of Pharmaceutical Sciences, Southern Medical University, Guangzhou, China; ^4^Faculty of Medical Sciences, University of Toronto, Toronto, Canada; ^5^School of Pharmacy, Pharmacy Australia Centre of Excellence (PACE), The University of Queensland, 20 Cornwall St., Woolloongabba, QLD 4102, Australia; ^6^Douglas Hospital Research Center, McGill University, Montreal, Canada

## Abstract

Glutamate-induced neurotoxicity is involved in various neuronal diseases, such as Alzheimer's disease. We have previously reported that glutamate attenuated the survival signaling of insulin-like growth factor-1 (IGF-1) by N-methyl-D-aspartate receptors (NMDARs) in cultured cortical neurons, which is viewed as a novel mechanism of glutamate-induced neurotoxicity. However, the phosphorylation sites of IGF-1 receptor (IGF-1R) affected by glutamate remain to be elucidated, and importantly, which subtype of NMDARs plays a major role in attenuating the prosurvival effect of IGF-1 is still unknown. In the present study, glutamate was found to attenuate the tyrosine phosphorylation of the IGF-1R and the prosurvival effect of IGF-1 in primary cultured cortical neurons. NMDAR inhibitors, MK801 and AP-5, blocked the inhibitory effect of glutamate on the phosphorylation of IGF-1R and increased cell survival, while DNQX, LY341495, and CPCCOEt had no effect. Interestingly, we found that glutamate decreased the phosphorylation of tyrosine residues 1131, 1135/1136, 1250/1251, and 1316, while it had no effect on tyrosine 950 in cortical neurons. Moreover, using specific antagonists and siRNA to downregulate individual NMDAR subunits, we found that the activation of NR2B-containing NMDARs was essential for glutamate to inhibit IGF-1 signaling. These findings indicate that the glutamate-induced attenuation of IGF-1 signaling is mediated by NR2B-containing NMDARs. Our study also proposes a novel mechanism of altering neurotrophic factor signaling by the activation of NMDARs.

## 1. Introduction

In mammalian brains, glutamate is an excitatory neurotransmitter that is critical for maintaining normal brain functions. However, under pathological conditions, highly activated glutamate receptors promote neuronal cell death and cause reactions from acute brain injury to chronic neurodegenerative diseases like Alzheimer's disease [1, 2]. Glutamate receptors include ionotropic and metabotropic receptors. The former have three different subtypes on the basis of their ligand-binding properties and sequence similarity: N-methyl-D-aspartate (NMDA) receptors, *α*-amino-3-hydroxy-5-methyl-4-isoxazole propionate (AMPA) receptors, and kainate receptors. The NMDA subtype (NMDAR) is an important class of ionotropic receptors that play essential biological functions in neurodevelopment, neural plasticity, and neuronal excitotoxicity [3, 4]. Under physiological condition, normal amounts of glutamate and appropriate activation of NMDAR are essential for neural communication, learning process, and memory formation [5]. In contrast, many pathological conditions may lead to the change of NMDAR expression or function [6]. The NMDAR is a heterooligomeric structure composed of the NR1 subunit, NR2A-D subunits, and the less common NR3A-B subunits. While the NR1 subunit is necessary for the formation and structure of NMDAR channels, the NR2 subunits determine the functional properties of these receptors. The NR2 and NR1 subunits are combined to form different heteromeric configurations, including NR1/NR2A, NR1/NR2B, and NR1/NR2A/NR2B combinations [7, 8]. NMDARs with different NR2 subunits may exhibit significantly different physiological and pathological functions. Furthermore, NMDAR subtypes containing NR2A and NR2B have different or opposing roles in modulating synaptic and neuronal plasticity [9]. For example, spike-timing-dependent potentiation induced by pairing pre- and postsynaptic spikes is mainly regulated by NMDARs containing NR2A subtype. In contrast, the NR2B subunit of NMDARs is involved in spike-timing-dependent depression [10]. Also, in the visual cortex, the ratio of NR2A to NR2B subunits may determine the normal formation of ocular plasticity [11]. Since these subtypes show differences in various processes, it is essential to address which subtype plays a vital role in specific physiological or pathological conditions.

Insulin-like growth factor 1 receptor (IGF-1R) signaling mediated by IGF-1 is an important pathway for the survival and maintenance of multiple cell types within the central nervous system [12]. Our previous studies showed that IGF-1 can protect neuronal cells from various insults [13, 14]. The physiological function of IGF-1 is mainly mediated by its highly-affinity receptors [15]. The binding of IGF-1 to IGF-1R leads to autophosphorylation and subsequent activation of its receptors, which subsequently activate various downstream substrates. Tyrosine phosphorylation of Shc by IGF-1R leads to the activation of the mitogen-activated protein kinase (MEK)/extracellular regulated protein kinase (ERK) and the phosphatidylinositide 3-kinase (PI3K)/protein kinase B (Akt) pathways [13]. It has been demonstrated that tyrosine residues 1131, 1135, and 1136 in the kinase domain of IGF-1R are initially phosphorylated, and the activation of these residues is necessary for the full activation of IGF-1R [16]. Tyr950 is the major binding site of Shc, which is the major substrate for IGF-1R. Through binding and phosphorylation of IRS-1 and IRS-2 and subsequent recruitment of Grb2 and PI3K regulatory subunit p85 to activate the Ras/MAPK and PI3K pathways, cell growth and survival are promoted [17]. Moreover, a study showed that under the stimulation of IGF-1, cells transfected with IGF-1R activate the signal transducer and activator of transcription 3 (STAT3). The possible mechanism of action is likely to be related to the fact that the activated receptor binds to Janus kinases (JAKs) and phosphorylates STAT3, thus participating in cell proliferation and differentiation [18, 19].

Our previous studies have demonstrated that treatment with glutamate impaired IGF-1R signaling in hippocampal neurons by activating NMDARs [20]. However, it is unclear which NMDAR subtype is involved. Therefore, in this study, we investigated the effect of glutamate on different phosphorylation sites of IGF-1R and the role of NMDAR subtypes in the effect of glutamate in primary cortical culture neurons. Our results indicate that the NR2B subunit plays a key role in the inhibition of glutamate on IGF-1R signaling. These findings provide a novel mechanism for explaining the inhibitory effect of glutamate on neurotrophic signaling which may be helpful for the understanding and treatment of several neurodegenerative diseases.

## 2. Materials and Methods

### 2.1. Materials

Human recombinant IGF-1 was obtained from Genentech Inc. (San Francisco, CA). NVP-AAM077 was generously provided by Y.P. Auberson, Novartis Pharma AG, Basel, Switzerland. Ro25-6981 was provided by the Department of Pharmaceutical Research Basel, F. Hoffmann-La Roche Ltd. 6,7-Dinitroquinoxaline-2,3-dione (DNQX), CPCCOEt, AP-5, and LY341495 were purchased from Tocris Bioscience Inc. (Minneapolis, Minnesota, USA). Dizocilpine maleate (MK-801), 3-(4,5-dimethylthiazol-2-yl)-2,5-diphenyl tetrazolium bromide (MTT), EDTA, and DMSO were purchased from Sigma-Aldrich (St. Louis, Missouri, USA). Anti-phospho-IGF-1R (Tyr1135/1136), anti-phospho-IGF-1R (Tyr1250/1251), anti-phospho-IGF-1R (Tyr1131), anti-phospho-IGF-1R (Tyr1316), anti-phospho-IGF-1R (Tyr950), anti-IGF-1R, anti-phospho-Akt (Ser473), anti-phospho-Akt (Thr308), anti-phospho-PDK1 (3-phosphoinositide-dependent protein kinase 1), anti-phospho-MEK, and anti-phospho-ERK1/2 antibodies were bought from Signalway Antibody Co. Ltd. (College Park, Maryland, USA). Anti-GAPDH was purchased from Cell Signaling Technology (Woburn, USA). All the primary antibodies used in this article were diluted at 1 : 1000. Secondary antibodies conjugated to horseradish peroxidase were bought from Santa Cruz Biotechnology (Santa Cruz, CA). Glutamate was obtained from Invitrogen (Carlsbad, CA, USA). Dulbecco's modified Eagle's medium (DMEM) and fetal bovine serum (FBS) were purchased from Gibco-BRL (NY, USA). 96-well shuttle rat neuron Nucleofector™ kits were purchased from Amaxa (Cologne, Germany).

### 2.2. Culture of Cortical Neurons

All experimental procedures were carried out following the NIH Guidelines for the Care and Use of Laboratory Animals, and the experimental protocols were approved by the Animal Care and Use Ethics Committee of the Sun Yat-sen University. Primary neuronal cultures were prepared from fetuses (embryonic day 16) obtained from pregnant Kunming mice (Experimental Animal Center, Sun Yat-sen University, Guangzhou, China) and cultured as described previously with minor modifications [20, 21]. Briefly, the mouse brains were dissected with scissors and the cerebral cortex was removed with forceps. After removal, tissues were washed 3 times with Ca^2+^- and Mg^2+^-free Hanks' balanced salt solution (HBSS) to remove the blood. The tissue was then cut into 1 mm^3^ size samples, washed three times with HBSS followed by digestion with 5 mL 0.25% trypsin at 37° C for 10 min. The digestion was stopped by the addition of 5 mL DMEM medium containing 10% fetal bovine serum. The tissue supernatant was collected and centrifuged at 1000 rpm for 5 min. The cell pellet was resuspended in neurobasal medium with 2% B-27, 2% HEPES, 0.25% Glutamax, and 1% antibiotic-antimyocotic. Cell density was determined by a cell counter. Cells were plated at a density of 5-8 × 10^5^ cells/mL in poly-D-lysine-coated culture plates. Neurons were kept in a 5% CO_2_-humidified atmosphere at 37°C. The experimental treatments were performed on the seventh day after plating.

### 2.3. Experimental Treatments

Before each experiment, the culture medium was replaced with neurobasal medium 2 h before adding the desired reagents. To study the signaling pathways induced by IGF-1 in cultured cortical neurons, cells were treated with 100 ng/mL IGF-1 for 8 min. Alternatively, cells were first exposed to either Ro25-6981 (1 *μ*M), NVP-AAM077 (1 *μ*M), MK801 (20 *μ*M), DNQX (10 *μ*M), AP-5 (10 *μ*M), CPCCOEt (10 *μ*M) or LY341495 (20 *μ*M) for 30 min followed by treatment with 1 mM glutamate for 1 h and then stimulation with 100 ng/mL IGF-1. All the experiments were repeated at least three times.

### 2.4. Western Blotting

Western blotting was carried out as described previously with minor modifications [22]. Briefly, cultured neurons were rinsed twice with ice-cold HBSS and lysed in RIPA buffer containing protease inhibitor cocktail and phosphatase inhibitors. Protein concentration was analyzed by BCA kit, and samples with equal amounts of protein were then separated by 4-20% SDS-PAGE and transferred to polyvinylidene fluoride membranes. To block the nonspecific binding, membranes were incubated with 5% nonfat milk and 2% bovine serum albumin in TBST for 1 h at room temperature. The membranes were then incubated overnight with primary antibodies at 4°C. The phosphorylation of IGF-1R, Akt, PDK-1, MEK, and ERK1/2 was determined by western blotting using anti-phospho-IGF-1R, Akt, PDK, MEK, and ERK1/2 antibodies, respectively. After washing, membranes were subsequently incubated with secondary antibodies at room temperature for 1 h. Membranes were finally washed several times with TBST to remove unbound secondary antibodies, and the bands were visualized using an ECL detection kit (Amersham Co, Toronto, ON). ImageJ was used to analyze the western blots.

### 2.5. Transfection of Cultured Cortical Neurons with siRNA for NR2A and NR2B by Nucleofector^™^

Primary cortical neurons were obtained as described above, and the transfection of siRNA for NR2A and NR2B was performed by a Nucleofector (Amaxa, Germany) with a 96-well shuttle rat neuron Nucleofector™ kit according to the manufacturer's instructions. Briefly, 2.5 × 10^5^ cells per sample were centrifuged at 80 × g for 10 min at room temperature. The supernatant was completely removed, and the cell pellets were resuspended in 20 *μ*L 96-well Nucleofector™ solution. Afterwards, 2 *μ*L NR2A-siRNA or NR2B-siRNA (300 nM) was added to each group and 20 *μ*L of cells with substrates was transferred into the wells of the 96-well Nucleocuvette™ Modules. The Nucleocuvette™ plate was gently taped to assure that the sample covered the bottom of the wells and placed in the 96-well Shuttle™. After run completion the cells were resuspended with 80 *μ*L prewarmed media and incubated in a humidified 37°C/5% CO_2_ incubator for another 48 h.

### 2.6. MTT Assay

Cells were pretreated with different blockers and inhibitors, including Ro25-6981 (1 *μ*M), NVP-AAM077 (1 *μ*M), MK801 (20 *μ*M), DNQX (10 *μ*M), AP-5 (10 *μ*M), CPCCOEt (10 *μ*M), or LY341495 (20 *μ*M) for 30 min and followed by treatment with 1 mM glutamate for 1 h and then stimulation with 100 ng/ml IGF-1 for 48 h. For the MTT assay, the medium was replaced with 0.5 mg/mL MTT in blank DMEM medium, and cells were incubated for another 4 h. After incubation, the MTT formazan crystals were solubilized by 100 *μ*L DMSO. The plates were placed on an orbital shaker for 10 min, and the survival profile of these cells was obtained spectrophotometrically by measuring the absorbance at 570 nm. Assays were repeated at least three times.

### 2.7. Statistical Analysis

Statistical analysis was performed using GraphPad Prism 5.0 statistical software (GraphPad software, Inc., San Diego, CA, USA). Data are expressed as the mean ± standard deviation (SD). Statistical analysis was carried out using one-way ANOVA followed by Tukey's multiple comparison, with *p* < 0.05 considered statistically significant. Using the two-tailed test, three samples per group were needed to detect a difference with 95% confidence and 80% power.

## 3. Results

### 3.1. Glutamate Attenuated IGF-1-Induced Tyrosine Phosphorylation of IGF-1 Receptors and Survival Effects of IGF-1 in Cultured Cortical Neurons

**Figure 1:**
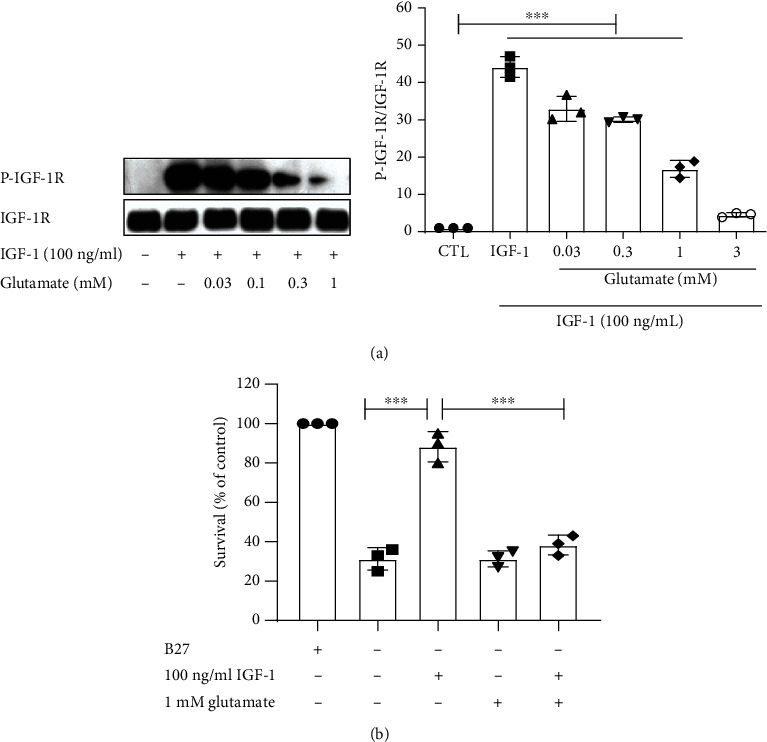
Glutamate decreases tyrosine phosphorylation of the IGF-1R and the prosurvival effect of IGF-1 in cultured cortical neurons. (a) Primary cultured cortical neurons were pretreated with 1 mM glutamate for 1 h and then exposed to 100 ng/ml IGF-1 for 8 min. Glutamate blocked the tyrosine phosphorylation of IGF-1R in a dose-dependent manner. Data represent assays from at least three independent experiments. (b) Cultured cortical neurons were pretreated with 1 mM glutamate, and then, cells were exposed to 100 ng/ml IGF-1 for 48 h and the cell viability was determined. Glutamate inhibited the prosurvival effect of IGF-1 in cultured cortical neurons. ^∗∗∗^*p* < 0.001; *n* = 3 independent experiments.

In our previous studies, we have proved that glutamate decreased the tyrosine phosphorylation of IGF-1R induced by IGF-1 in cultured hippocampal neurons from Sprague-Dawley rats. Simultaneously, glutamate attenuated the protective effect of IGF-1 [20]. To confirm this effect and lay the foundation for the subsequent experiments, we first investigated the effect of glutamate on IGF-1R signaling and its prosurvival properties in cultured cortical neurons. Obtained results show that treatment with IGF-1 led to a significant tyrosine phosphorylation of IGF-1R, while glutamate inhibited the tyrosine phosphorylation of IGF-1 receptor in cultured cortical neurons (Figure 1(a)). This inhibition was observed beginning at the concentration of glutamate of 0.03 mM and being maximal at 1 mM. We then evaluated whether glutamate was able to block the prosurvival effects of IGF-1. B27 was used as a positive control, and as shown in Figure 1(b), decreased cell viability was observed in B27-deprived neurons. To exclude the influence of B27 on the prosurvival effect of IGF-1, neurons treated with IGF-1 were deprived of B27, and cell viability was determined by MTT assay. Similarly, glutamate blocked the prosurvival effect of IGF-1 in cortical cultured neurons (Figure 1(b)). These results demonstrate that glutamate is able to attenuate the tyrosine phosphorylation of the IGF-1R and IGF-1-mediated survival effect in cultured cortical neurons.

### 3.2. The Effect of Glutamate on Different Tyrosine Residues of the IGF-1R

**Figure 2:**
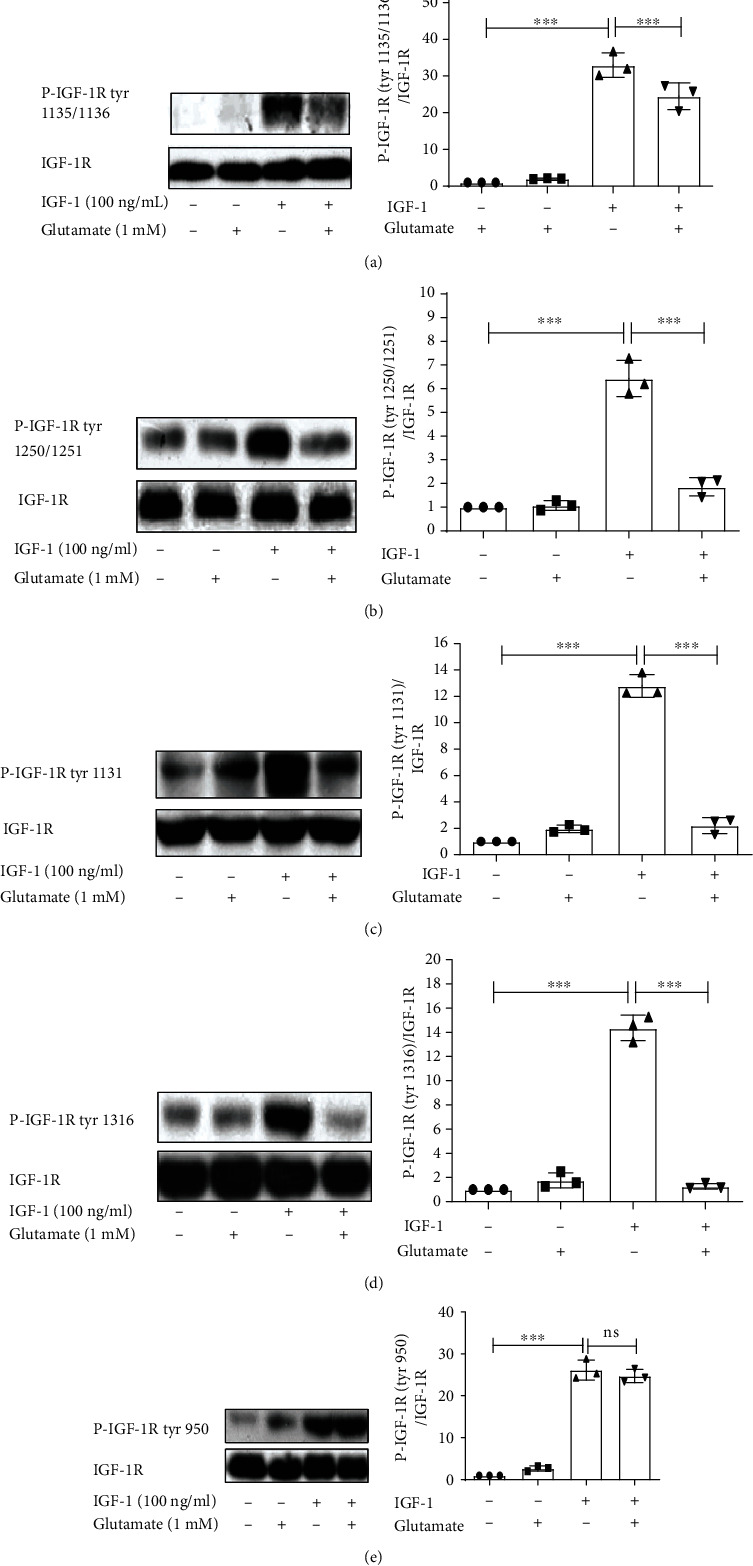
The effect of glutamate on different IGF-1R tyrosine residues. Following pretreatment with 1 mM glutamate, cultured cortical neurons were exposed to 100 ng/ml IGF-1 for 8 min (for tyrosine phosphorylation of IGF-1R). Different primary antibodies of specific tyrosine residues of IGF-1R were used to test the influence of glutamate on IGF-1R. Data represent assays from at least three independent experiments. ^∗∗∗^*p* < 0.001.

Knowing that glutamate is able to attenuate tyrosine phosphorylation of IGF-1R and the survival effect of IGF-1 in cultured cortical neurons, we further investigated the effect of glutamate on the different phosphorylation sites of IGF-1Rs. For this purpose, antibodies against anti-phospho-IGF-1R (Tyr1135/1136), anti-phospho-IGF-1R (Tyr1250/1251), anti-phospho-IGF-1R (Tyr1131), anti-phospho-IGF-1R (Tyr1316), and anti-phospho-IGF-1R (Tyr950) were used to detect the effect of glutamate on the abovementioned phosphorylation sites. The results show that IGF-1 significantly increased the phosphorylation of IGF-1R at Tyr950, 1135/1136, Tyr1250/1251, Tyr1131, and Tyr1316. Glutamate attenuated the phosphorylation of IGF-1R at Tyr1135/1136, Tyr1250/1251, Tyr1131, and Tyr1316, while it had no effect on the phosphorylation at Tyr950 (Figure 2). As tyrosine residues 1131, 1135, and 1136 play an essential role in the activation of IGF-1R, these results indicate that glutamate inhibited the activation of IGF-1R, even though Tyr950 is not involved.

### 3.3. Glutamate Attenuated the Survival/Protective Effect of IGF-1 in Cultured Cortical Neurons through NMDARs

**Figure 3:**
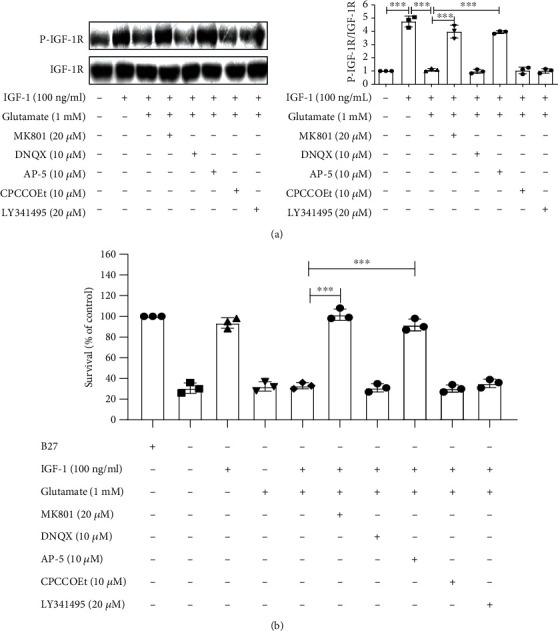
Glutamate attenuates tyrosine phosphorylation and the survival/protective effect of IGF-1R through NMDARs. (a) Cultured cortical neurons were pretreated with the NMDA receptor antagonists MK801 or AP-5 (two selective NMDA receptor antagonists), DNQX (antagonist for the non-NMDA receptor, AMPA, and kainate receptors), CPCCOEt (selective, non-competitive mGluI antagonist), or LY341495 (mGluIII/II antagonist), and the effect of glutamate (1 mM) on IGF-1R phosphorylation was measured. Data represent assays from at least three independent experiments. (b) Cultured cortical neurons were pretreated with the NMDA receptor antagonists MK801 or AP-5, DNQX, CPCCOEt, or LY341495, and the effect of glutamate (1 mM) on the survival effect of IGF-1 was measured. Data represent assays from at least three independent experiments. ^∗∗∗^*p* < 0.001.

The biological functions of glutamate are achieved through two types of receptors: ionotropic receptors, also known as ligand-gated ion channel, and G-protein-coupled receptors, known as metabotropic receptors. In order to determine the involved glutamate receptor subtype, cultured cortical neurons were pretreated with various inhibitors specific of the different glutamate receptor subtypes. Specifically, we used the following: (1) selective NMDA receptor antagonists, MK801, or AP-5; (2) antagonists for non-NMDA receptors, AMPA and kainite receptors, and DNQX; (3) selective, noncompetitive mGluI antagonist, CPCCOEt; and (4) mGluR antagonist LY341495. After treatment with these blockers or antagonists, the effect of glutamate (1 mM) on the phosphorylation of IGF-1R and its related signaling molecules was determined by western blot. The results reveal that MK801 (20 *μ*M) reversed the inhibitory effect of glutamate on the tyrosine phosphorylation of IGF-1R induced by IGF-1 in cortical cultured neurons. By comparison, AP-5 also blocked the effect of glutamate. In contrast, DNQX, at a concentration (20 *μ*M) known to block non-NMDA receptors, had no effect (Figure 3(a)). The metabotropic glutamate receptor subtype antagonists, CPCCOEt and LY341495, had no effect on the inhibitory effect of glutamate on IGF-1 signaling (Figure 3(a)). Moreover, IGF-1 protected cultured cortical neurons from B27 deprivation-induced cell death, glutamate attenuated the survival promoting effect of IGF-1, and MK801 reversed the effect of glutamate (Figure 3(b)). Similar results were obtained in PC12 cells (Fig. S1 and Fig. S2). Consistent with our previous results in hippocampal neurons, these results confirm that glutamate is able to block the survival signaling of IGF-1 by activating NMDA receptors in cortical neurons.

### 3.4. Glutamate Inhibited IGF-1-Induced Activation of the PI3K/Akt Pathway, while Enhancing the Effect of IGF-1 on ERK

**Figure 4:**
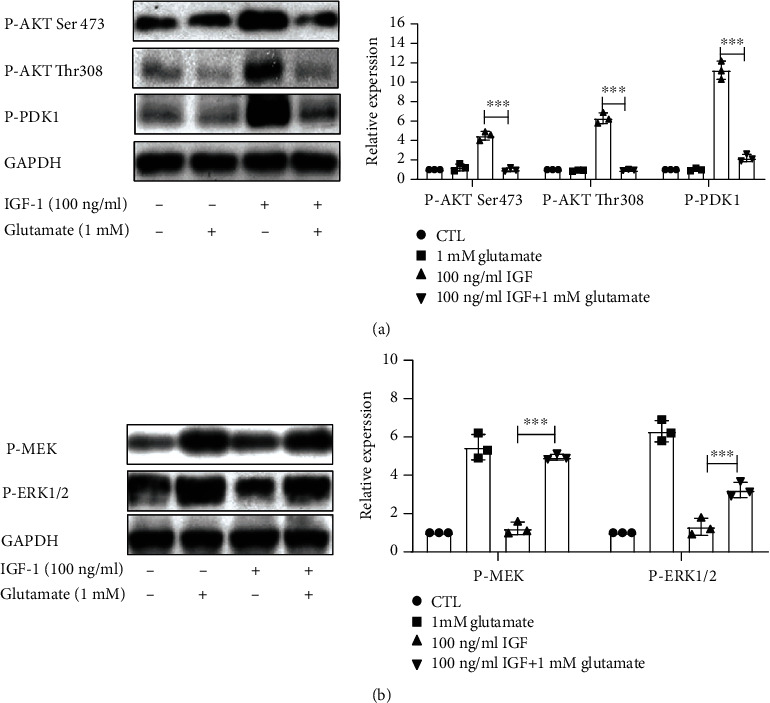
Glutamate inhibits IGF-1-induced activation of the PI3K/Akt pathway, while enhancing the effect of IGF-1 on ERK. Cultured cortical neurons were pretreated with glutamate (1 mM) for 1 h and then exposed to 100 ng/ml IGF-1 for 8 min. Determination of (a) AKT and PDK1 phosphorylation levels and (b) MEK and ERK1/2 phosphorylation levels by western blot. Blots represent prototypical examples of experiments replicated at least three times. ^∗∗∗^*p* < 0.001.

Phosphorylation of the IGF-1R is a particularly important survival-promoting signal. The PI3K/Akt and MEK/MAPK signal pathways are two key signaling pathways downstream of the IGF-1R. Hence, we investigated the role of glutamate on the phosphorylation of PDK1 (the upstream kinase of Akt), Akt, MEK, and ERK1/2. As shown in Figure 4(a), pretreatment with glutamate (1 mM) attenuated the phosphorylation of Akt (both Thr-308 and Ser-473) and inhibited the activation of PDK1 (Figure 4(a)). On the contrary, glutamate enhanced IGF-1-induced activation of ERK1/2 and its upstream kinase MEK (Figure 4(b)). Taken together, these results indicate that glutamate not only suppresses the activation of IGF-1R but also inhibits IGF-1R-mediated downstream signaling, while having different effects on PI3K/Akt and MEK/ERK1/2 signaling in cortical neurons treated with IGF-1.

### 3.5. Glutamate Inhibited the Tyrosine Phosphorylation of IGF-1R and the Survival Effect of IGF-1 via NR2B-Containing NMDARs

**Figure 5:**
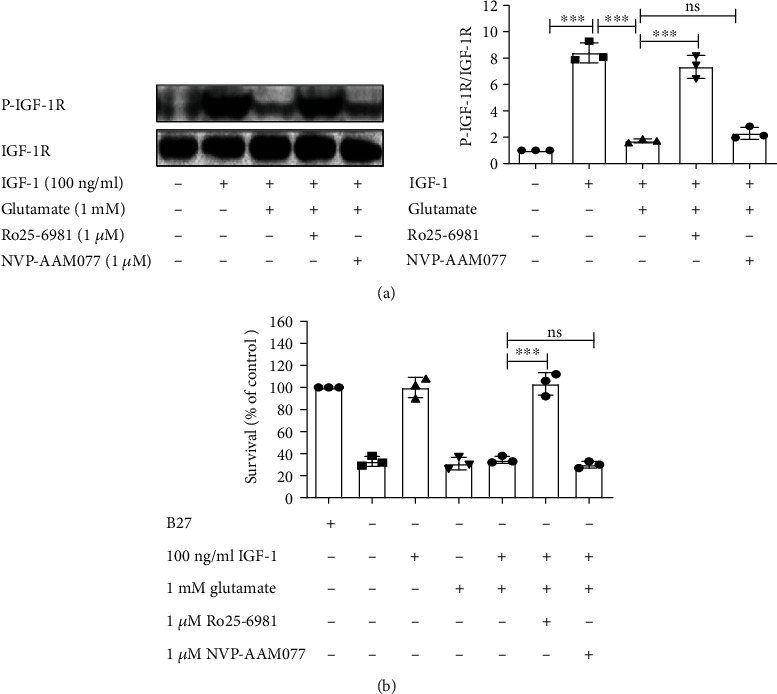
NR2B antagonist hinders the inhibitory effect of glutamate on the tyrosine phosphorylation of IGF-1R and the survival effect of IGF-1 on cultured cortical neurons. Following pretreatment with Ro25-6981 (1 *μ*M) and NVP-AAM077 (1 *μ*M) for 30 min, 1 mM glutamate was given to cultured cortical neurons. Then, the neurons were exposed to 100 ng/ml IGF-1 for 8 min (for tyrosine phosphorylation of IGF-1R) and 48 h (for survival assay). The tyrosine phosphorylation of the IGF-1R, densitometric analysis (a), and cell viability (b) were determined as described in Experimental Treatments. Ro25-6981 blocked the effect of glutamate on the tyrosine phosphorylation of the IGF-1R (a) and protected cortical neurons from glutamate's excitotoxicity (b). Blots represent prototypical examples of experiments replicated at least three times. Data represent assays from at least three independent experiments. ^∗∗∗^*p* < 0.001.

**Figure 6:**
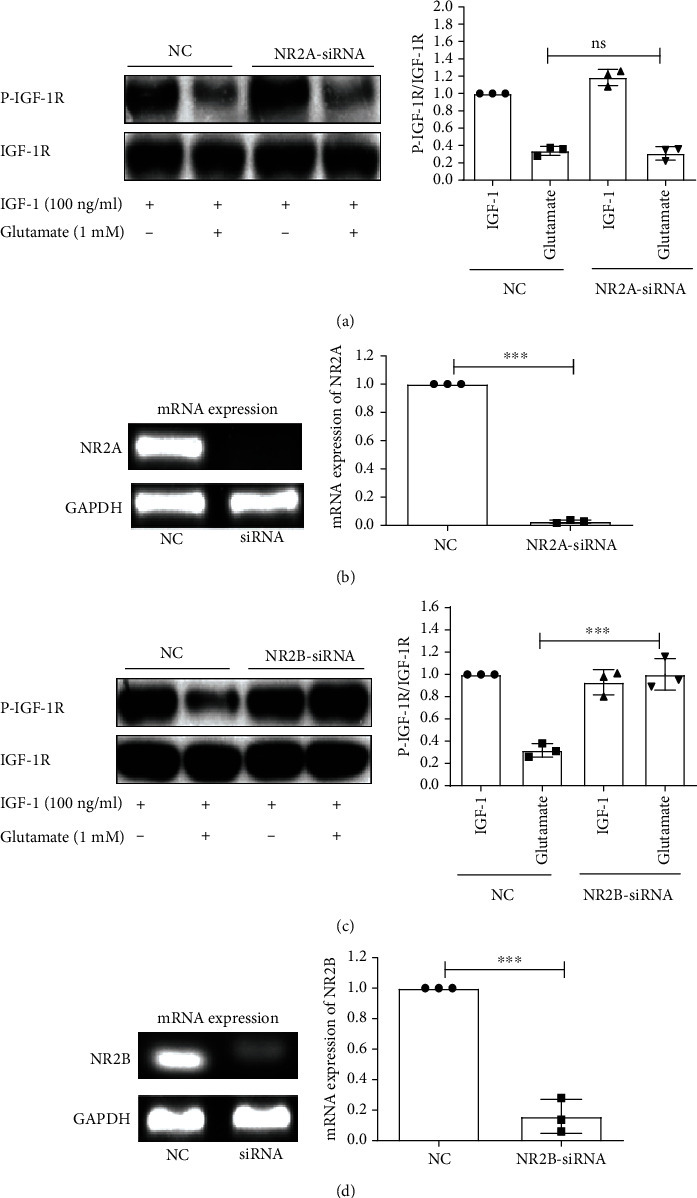
NR2B-containing NMDARs are responsible for the inhibitory effect of glutamate on the tyrosine phosphorylation of IGF-1R. Following the siRNA, 1 mM glutamate was given to cultured cortical neurons. Then, the neurons were exposed to 100 ng/ml IGF-1 for 8 min. (a–c) The phosphorylation of IGF-1R was determined by western blot. (b–d) siRNA inhibited the expression of NR2A and NR2B. Blots represent prototypical examples of experiments replicated at least three times. Data represent assays from at least three independent experiments. ^∗∗∗^*p* < 0.001.

The functions of NMDARs are mainly affected by the different NR2 subunits. To test the difference between NR2A and NR2B subunits of the NMDAR in the regulation of glutamate-induced attenuation on IGF-1 signaling, we used subunit-specific NMDA receptor antagonists. These antagonists were NVPAAM077, which preferentially inhibits NR2A-containing receptors [23, 24], and Ro 25–6981, which specifically blocks NR2B-containing receptors [25, 26]. As shown in Figure 5(a), Ro25-6981 significantly blocked the inhibitory effect of glutamate on the phosphorylation of IGF-1R. Consistent with this result, Ro25-6981 also blocked the prosurvival property of IGF-1R (Figure 5(b)), while NVP-AAM077 had no effect. To further confirm the role of the NR2B and NR2A receptor subunits, siRNAs specific for NR2B and NR2A were transfected in cortical neurons using Nucleofector technology. Figures 6(b) and 6(d) show that the mRNA levels of NR2A and NR2B were significantly knocked down. We then investigated the effect of NR2B or NR2A knockdown on the phosphorylation of IGF-1R induced by IGF-1. The results revealed that the siRNA for NR2B blocked the effect of glutamate on the phosphorylation of IGF-1R (Figure 6(a)), while the siRNA for NR2A did not have an effect (Figure 6(c)). These results indicate that the NR2B subtype plays a major role in the inhibitory effect of glutamate on IGF-1R phosphorylation.

## 4. Discussion

Excessive extracellular glutamate concentration can cause uncontrolled and continuous depolarization of neurons causing excitotoxicity. In excitotoxicity, glutamate upregulates nNOS, mitochondrial dysfunction, ROS production, ER stress, and lysosomal enzyme release triggering apoptosis [27]. The present study proved that glutamate attenuated neuronal survival through the inhibition of IGF-1R signaling. Despite calcium entry through NMDA receptors being a well-known mediator of glutamate toxicity, obtained results provide a novel mechanism of glutamate toxicity, as we found that calcium influx was not involved in the inhibitory effects of glutamate on neuronal IGF-1 signaling (data not shown). Therefore, glutamate toxicity is likely to be due to both calcium overload and neuronal IGF-1 receptor impairments. Evidences suggest that IGF-IR signaling is required for both survival and growth of many cell types [28, 29]. IGF-1 binds to its receptors initiating its autophosphorylation, which further triggers its intrinsic tyrosine kinase activity, resulting in the phosphorylation of many intracellular substrates, followed by the activation of several downstream signaling axes such as PI3K/AKT and MAPK/ERK1/2 pathways [30]. Our previous studies also showed that IGF-1 protected SH-SY5Y cells against serum deprivation insult and activated IGF-1R, AKT, and ERK1/2 signaling [31]. In contrast, glutamate is a neurotoxic neurotransmitter, whose excitotoxic mechanisms are due to energy failure, oxidative stress, calcium overload, and subsequent apoptosis [32, 33]. In this study, we aimed to examine the role of glutamate in IGF-1-induced IGF-1R phosphorylation and its downstream signaling pathways. We found that glutamate not only activates the classical pathways mentioned above leading to cell death but also inhibits prosurvival pathways mediated by IGF-1R, unravelling a possible novel mechanism for the toxicity of glutamate in the central nervous system. IGF-1R autophosphorylation is the initial and necessary proximal step for its activation. As IGF-1 induces multiple phosphorylation sites in IGF-1R, in this study, we performed for the first time a systematic evaluation of the effect of glutamate on the activation of IGF-1R at different phosphorylation sites and found that glutamate attenuates the phosphorylation of IGF-1R at Tyr1135/1136, Tyr1250/1251, Tyr1131, and Tyr1316, with no effect on the phosphorylation at Tyr950. This result is indicative that glutamate may have various effects on different phosphorylation sites of IGF-1 and may have an alternative effect on IGF-1 signaling and biological functions as it was reported that special phosphorylation sites of IGF-1R are linked to its various signaling and functions [34]. Consistent with this, the study of the effect of glutamate on individual downstream signaling of IGF-1R showed different effects of glutamate in IGF-1R signaling. Specifically, glutamate inhibited the PI3K/Akt signal pathway which is the major survival signaling pathway in primary culture neurons supporting the results obtained in the survival assay in which glutamate was shown to attenuate the survival protection of IGF-1. In contrast, glutamate enhanced the phosphorylation of MEK/ERK1/2. It has been reported that sustained activation of ERK1/2 signaling plays a proapoptotic role in glutamate-induced death of astrocytes [35]. But also, studies report that MEK/ERK1/2 can promote cell survival by enhancing the activity of antiapoptotic molecules [36]. However, the detailed mechanisms need further investigation in a future study. Taken together, obtained results suggest that the prosurvival effect of IGF-1 in primary cortical neurons may be through the PI3K/Akt and MEK/ERK1/2 signaling pathway.

Excessive activation of NMDARs causes excitotoxicity, which has been implicated in the inhibition of neurotrophic factor signaling and consequently to a wide variety of neurodegenerative disorders [37]. Extrasynaptic NMDAR activation and dysfunctional BDNF signaling are early hallmarks of neurodegenerative diseases such as Alzheimer's disease (AD), Huntington's disease (HD), and Parkinson's disease (PD) [38]. Moreover, cell death triggered by seizure or ischemic stroke is also attributed to NMDAR overactivation [39]. Since the function of NMDARs is greatly influenced by their component subunits, especially the NR2 subunits, we investigated the influence of different NR2 subunits on IGF-1 signaling. Using subunit-specific NMDAR antagonists, we found that the NR2B subunits of NMDARs are responsible for the inhibitory effect of glutamate on the tyrosine phosphorylation and prosurvival actions of the IGF-1R, while NR2A subunits exhibited little effect. Further confirmation using siRNA revealed that the siRNA for NR2B blocked the effect of glutamate on the phosphorylation of IGF-1R, while siRNA for NR2A had no effect. Similar results have also been obtained in cortical neurons, whose NR2A subunits of NMDA receptors promote the trafficking of GluR1, while NR2B subunits of NMDA receptors inhibit the trafficking of GluR1 [40]. Thus, these two subunits contribute differentially to the function of different signaling proteins. Moreover, many reports have suggested that different NMDAR subtypes, including NR2A and NR2B subunits, have different or even opposing roles in governing the direction of neural plasticity and in mediating NMDA-elicited neuronal survival and cell death [41, 42]. In addition to NR1/NR2A and NR1/NR2B heterodimers, cortical neurons also express an NR1/NR2A/NR2B heterotrimeric NMDAR subpopulation. However, no selective antagonist for these receptors has been found; hence, their contribution to NMDAR function is not clear. Nevertheless, NVP-AAM007 has been claimed to be an inhibitor of NR2A-contaning NMDA receptors and could be used as a selective antagonist for this purpose [43]. Furthermore, because NVP-AAM077 is a competitive antagonist at the agonist binding sites of NR2A subunits and both of the binding sides of NR1/NR2A/NR2B and NR1/NR2A receptors need to be occupied to open the channel, we speculate that NVP-AAM077 may inhibit all NR2A subunits of NMDARs. On the other hand, Ro 25–6981 has been demonstrated to have a higher selectivity towards the NR1/NR2B heterodimeric receptors than the NR1/NR2A/NR2B heterotrimeric receptors [44]. Thus, considering the existence of NR1/NR2A/NR2B heterotrimeric receptors and the lack of a selective antagonist, we tentatively classified the NVP-AAM077 and Ro 25-6891 compounds as antagonists towards NR2A and NR2B subunits of NMDARs, respectively. Taken together, our results provide an explanation for the different roles of NR2B and NR2A subunits of NMDARs in regulating the effect of glutamate on IGF-1R.

## 5. Conclusion

**Figure 7:**
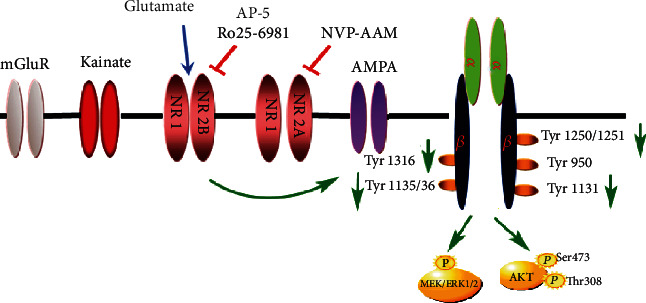
Possible mechanism of action of glutamate on IGF signaling. Glutamate stimulates the activation of NMDARs, leading to the activation of NR2B subunits and the release of calcium ions. The overloaded calcium ions act on the IGF-1R. We used a variety of highly selective inhibitors and siRNA technology for key research targets to verify the conclusions of this study.

In summary, the present study demonstrates that glutamate attenuates tyrosine phosphorylation of the IGF-1R and cell viability via the NR2B subunit of NMDARs. These findings provide a novel mechanism for the action of glutamate on IGF-1 signaling in neurons (Figure 7). Glutamate can regulate neuronal viability by altering trophic factor receptor signaling and the downstream substrates, including PI3K/Akt and MEK/ERK1/2 pathways. These observations may be helpful in understanding the mechanisms of action of glutamate on neuronal damage from acute ischemic brain injuries to chronic neurodegenerative diseases.

## Data Availability

The data used to support the findings of this study are included within the article.

## Supplementary Materials

Supplementary Materials Fig.s1: glutamate inhibited the prosurvival effect of IGF-1 in PC12 cells. PC12 cells were pretreated with 1 mM glutamate for 1 h, and then, cells were exposed to 100 ng/ml IGF-1 for 48 h and the cell viability was determined. Fig.s2: glutamate attenuates the cell viability of IGF-1R through NMDARs. PC12 cells were pretreated with the NMDA receptor antagonists MK801 or AP-5 and CPCCOEt or LY341495, and the effect of glutamate (1 mM) on the survival effect of IGF-1 was measured. Data represent assays from at least three independent experiments.
