# Supplementary material for: Glutamate Attenuates the Survival Property of IGFR through NR2B Containing N-Methyl-D-aspartate Receptors in Cortical Neurons
Source: Oxid Med Cell Longev. 2020 Aug 11;2020:5173184. doi: 10.1155/2020/5173184 (PMC7441446; doi:10.1155/2020/5173184)
Supplement: Supplementary Materials — Fig.s1: glutamate inhibited the prosurvival effect of IGF-1 in PC12 cells. PC12 cells were pretreated with 1 mM glutamate for 1 h, and then, cells were exposed to 100 ng/ml IGF-1 for 48 h and the cell viability was determined. Fig.s2: glutamate attenuates the cell viability of IGF-1R through NMDARs. PC12 cells were pretreated with the NMDA receptor antagonists MK801 or AP-5 and CPCCOEt or LY341495, and the effect of glutamate (1 mM) on the survival effect of IGF-1 was measured. Data represent assays from at least three independent experiments. [file 5173184.f1.pdf]

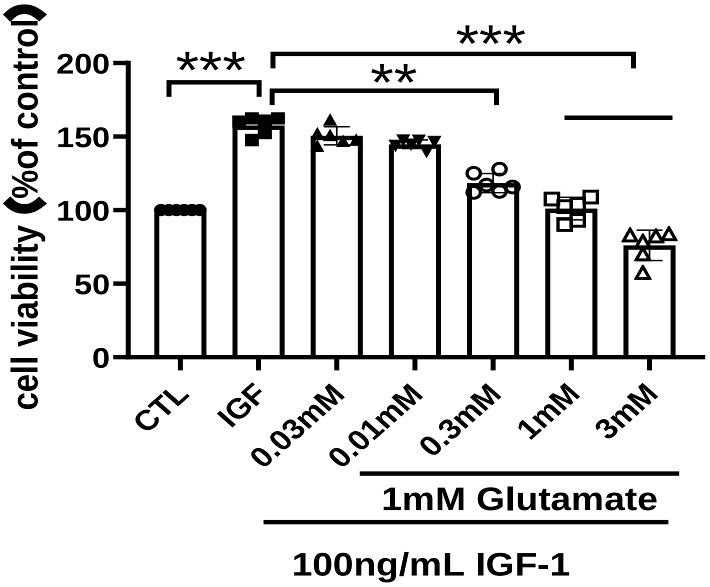

Fig.s1. Glutamate inhibited the pro-survival effect of IGF-1 in PC12 cells. PC12 cells were pretreated with 1 mM glutamate for 1h, and then cells were exposed to 100 ng/ml IGF-1 for 48 h and the cell viability was determined.

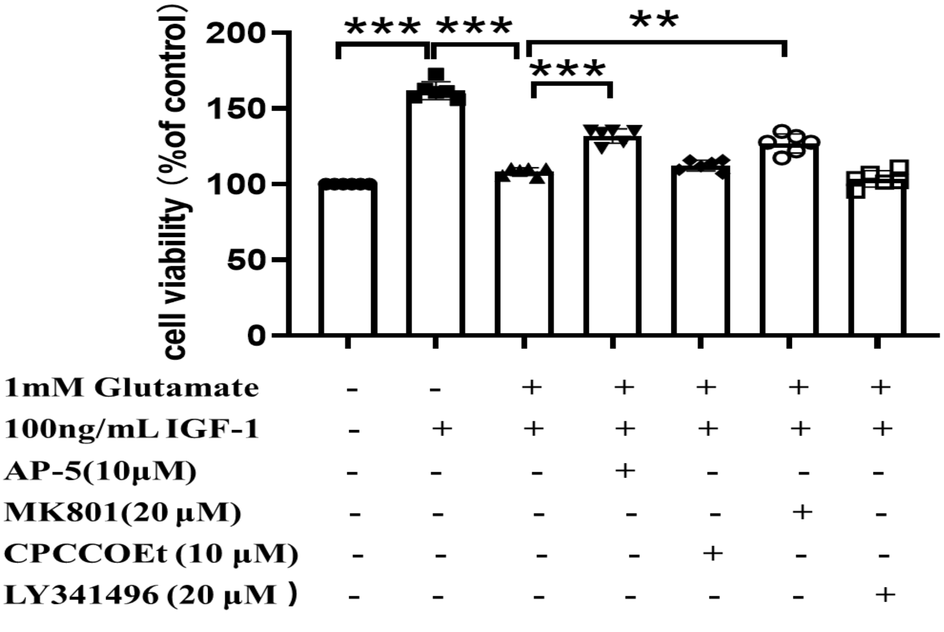

Fig.s2. Glutamate attenuates the cell viability of IGF-1R through NMDARs. PC12 cells were pretreated with the NMDA receptor antagonists MK801 or AP-5, CPCCOEt or LY 341496, and the effect of glutamate (1mM) on the survival effect of IGF-1 was measured. Data represent assays from at least three independent experiments.
